# Telerehabilitation and Face-to-Face Exergame Delivery Modalities to Improve Postural Control in Older Adults: A Randomised Controlled Trial

**DOI:** 10.3390/medsci13040270

**Published:** 2025-11-14

**Authors:** Valeska Gatica-Rojas, Ricardo Cartes-Velásquez, Javier Silva-Llanos, Catalina Arenas-Leiva, Valentina De Vitis, Simone Posella, Luis Eduardo Cofré Lizama

**Affiliations:** 1 Human Motor Control Laboratory, Faculty of Health Sciences, Universidad de Talca. Av. Lircay S/N, Talca 3460000, Chile; javier.silva@utalca.cl (J.S.-L.); catalina.arenas@utalca.cl (C.A.-L.); 2 Facultad de Derecho y Ciencias Sociales, Universidad San Sebastián, Concepción 4080871, Chile; cartesvelasquez@gmail.com; 3 OT Biolettronica, Via San Marino 21, 10134 Torino, Italy; v.devitis@otbioelettronica.it (V.D.V.); s.posella@otbioelettronica.it (S.P.); 4 Department of Allied Health, School of Health Sciences, Swinburne University of Technology, Hawthorn, Melbourne, VIC 3122, Australia; eduardocofre@swin.edu.au; 5 Department of Medicine (Royal Melbourne Hospital), The University of Melbourne, Melbourne, VIC 3052, Australia

**Keywords:** randomised clinical trial, telerehabilitation, older adults, postural control, virtual reality, exergame, physical therapy

## Abstract

Background: A systematic exercise programme using low-cost virtual reality devices can help maintain and improve postural control in older adults. This study aimed to evaluate the effectiveness of two different exergame programme modalities: telerehabilitation (TR) and face-to-face (FF). Methods: A randomised controlled trial was conducted with 16 participants aged 65 to 75. Both groups completed an 18-session exergame intervention over six weeks, with the TR group (exposure) receiving remote sessions and the FF group having in-person (control) sessions with a physiotherapist. Assessments were carried out at baseline, at weeks 2, 4, and 6, with two follow-ups at weeks 8 and 10. Centre of Pressure (CoP) measures in tasks: eyes open (EO), eyes closed (EC), medial-lateral (ML) weight-shifting exergame and anterior–posterior (AP) weight-shifting exergame, and clinical tests were used to evaluate postural control. Results: TR and FF significantly reduced the CoP*_Sway-area_* during EC (TR: *p* < 0.01; FF: *p* = 0.01) at 6 weeks and only FF demonstrated a significant reduction during EO (*p* < 0.01). Post hoc analysis revealed that TR maintained a significant reduction in the secondary outcomes of the CoP at 8 and 10 weeks, while FF did not sustain these effects over time. Between-group comparisons revealed a greater effect of TR in CoP*_Sway-area_*, and secondary outcomes during the AP weight-shifting task (*p* < 0.01) at 6 weeks, whereas the FF had a greater effect in secondary CoP outcomes during the ML weight-shifting task (*p* < 0.01) at 6 weeks. Conclusion: Both six-week exergame programmes were equally effective at improving postural control. Given the observed specific effects of TR and FF delivery, physiotherapists can consider either modality to suit individual needs and access, or as a complementary approach to maintain and improve postural control in older adults.

## 1. Introduction

Engaging in regular physical activity offers wide-ranging benefits for older adults, including the preservation of cognitive function, neuromotor coordination, and cardiopulmonary capacity [1,2,3]. These effects not only promote overall health but also support independence [4,5], social participation [5], and quality of life [6]. In contrast, physical inactivity is linked to adverse outcomes such as increased risk of falls, fractures, functional decline, and hospitalizations [4]. Among these, falls are a major public health concern [7], often stemming from age-related impairments in postural control [8,9]. This highlights the need for safe, effective, and accessible exercise interventions that support balance [5] and functional independence [4].

The COVID-19 pandemic accelerated the uptake of telemedicine, including telerehabilitation (TR), as a means of maintaining therapeutic engagement during periods of physical distancing [10,11]. Remote physiotherapy programmes targeting neuromotor function, particularly gait and balance, emerged as vital tools to support functional health while reducing healthcare costs and caregiver burden [12,13,14]. In this context, physiotherapists rapidly adapted their models of care from face-to-face (FF) delivery to remote formats, emphasising the need for scalable and sustainable digital approaches for older adults.

Recent systematic reviews indicate that physiotherapist-led, exercise-based TR is comparable in effectiveness to FF interventions and superior to no treatment [15], particularly in older adults with musculoskeletal and cardiopulmonary conditions [15]. These findings have driven the development of public policies and community-based exercise programmes supporting healthy ageing, although long-term sustainability remains a challenge, especially in low-resource settings.

One promising innovation in this space is the use of motion-based video games, or exergames, which engage users in multi-sensory, interactive exercises. Exergames have shown benefits for postural control, mobility, and quality of life in older adults [5,16] and those with neurodegenerative conditions [17]. However, access to advanced systems remains limited in underserved communities due to high equipment costs. To address this, researchers have explored affordable, commercially available solutions such as the Nintendo Wii, which features motion-sensitive controls and a balance board, and is widely available as a refurbished or second-hand device.

While exergames delivered in person under physiotherapist supervision have demonstrated effectiveness [18], it remains unclear whether the same interventions, when delivered synchronously via videoconferencing, are equally effective. As the demand for community-based physiotherapy grows, there is a pressing need to evaluate telerehabilitation models that are both accessible and clinically effective.

This study aimed to evaluate the effectiveness of a six-week, low-cost exergame programme delivered via synchronous videoconferencing TR versus FF therapy in older adults. Specifically, we sought to answer the research question: Is an exergame intervention using telerehabilitation as effective as face-to-face delivery for improving postural control in older adults? Given that both programmes delivered the same multi-sensory exergames and differed only in mode of delivery, we hypothesised that TR will be as effective as face-to-face. Demonstrating comparable effectiveness would support the adoption of TR as a viable, scalable, and physiotherapy-complementary option to maintain or improve postural control in older adults.

## 2. Materials and Methods

The study was conducted in accordance with the Decla-ration of Helsinki and approved by the University of Talca Ethics Committee, Chile (ID No. 24-2018, date of approval: 26 September 2018). All participants voluntarily signed an informed consent form, with the research explained in detail both verbally and in writing.

### 2.1. Study Design and Sample Size

**Figure 1:**
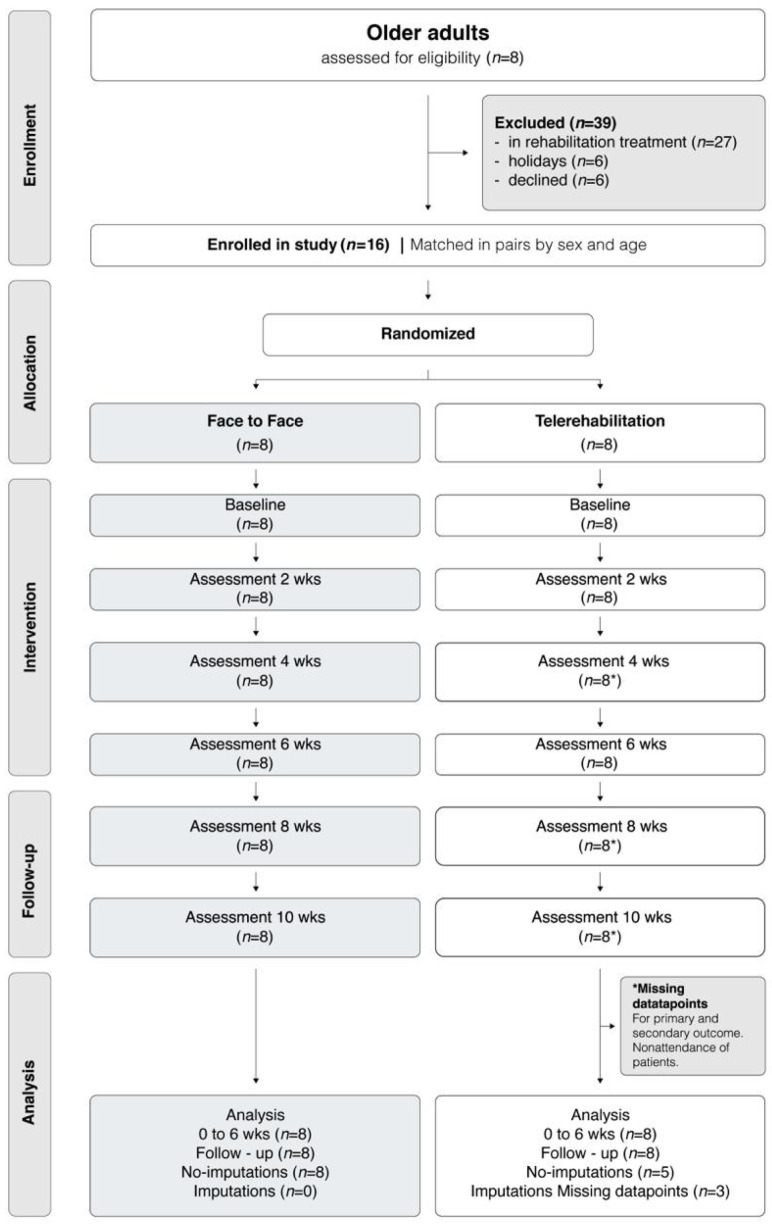
Flow diagram of the prospective, randomised controlled trial with matched-pairs allocation in older adults. wks = weeks. * Missing datapoints. Other options: Missing datapoints for primary and secondary outcome.

This was a two-arm, randomised controlled trial with concealed allocation, triple blinding, and intention-to-treat analysis. In the FF group, the physiotherapist was blinded by being instructed only to deliver the exergame intervention, without knowledge of the TR group’s existence. Similarly, the physiotherapist assisting the TR group was unaware of the FF group. Blinding was maintained by conducting TR and FF sessions in distant locations. The study protocol was prospectively registered and published to ensure transparency in data collection and analysis [19] (Figure 1). The interventions were described using the Template for Intervention Description and Replication (TIDieR) framework to enhance reproducibility and the trial is reported in accordance with the CONSORT guidelines [20].

Sample size was obtained from previous studies, and a between-group difference of 1.5 cm^2^ was considered clinically relevant [5,16,19,21]. Assuming a standard deviation of 1 cm^2^, an alpha of 0.05, 80% power, and a 5% attrition rate, a minimum of eight participants per group (TR and FF) were required. The sample size was calculated using GRANMO v.7.12 (Institut Municipal d’Investigació Mèdica, Barcelona, Spain).

### 2.2. Recruitment

Participants were recruited from two senior citizen clubs affiliated with community health centres in central Chile. Two physiotherapists and one medical doctor screened and enrolled participants who met the eligibility criteria until the required sample size was reached. Inclusion criteria were: (1) aged between 65 and 75 years, (2) Mini-Mental State Examination (MMSE) score > 24 points, (3) Short Physical Performance Battery (SPPB) score > 7 points and (4) no history of falls in the past 12 months. Exclusion criteria were: (1) vestibular impairment, (2) uncorrected visual impairments, (3) physiotherapy received within the past 6 months, and (4) prior experience using the Nintendo Wii.

The study followed the ethical principles outlined in the Declaration of Helsinki, the Belmont Report, and CIOMS Guidelines, and it adhered to relevant Chilean laws. Ethical approval was obtained from the University of Talca Ethics Committee (Ref. No. 24-2018), and the trial was registered with the Australian and New Zealand Clinical Trials Registry under the number ACTRN12621001380886, which is part of the International Clinical Trials Registry Platform (ICTRP). All methods were performed in accordance with these guidelines and regulations. All participants provided written informed consent prior to their involvement in the study.

### 2.3. Randomization

Participants were randomly assigned in a 1:1 ratio to two treatment groups, TR or FF intervention group, with randomization performed using a computerised, web-based system. After allocation, participants were informed of their group and received a detailed explanation of the intervention. To support adherence, periodic consultations were held to clarify intervention content. All outcome assessors were blinded to group allocation. Data entry was performed by an independent research assistant using confidential datasheets to preserve blinding during analysis. The study flowchart is shown in Figure 1.

### 2.4. Posturographic and Clinical Measurements

All postural and clinical measurements described below were performed at baseline, weeks 2 and 4 (mid-intervention), week 6 (end of the intervention), and weeks 8 and 10 (follow-ups). The total time for postural tasks and clinical tests per participant at each assessment week was 20 to 30 min.

#### 2.4.1. Postural Tasks

**Figure 2:**
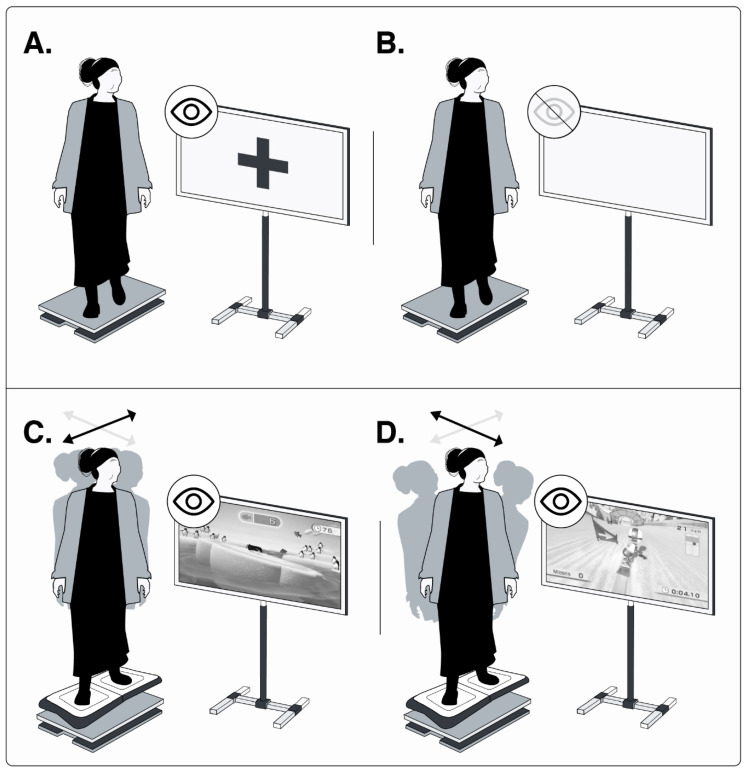
Four postural tasks. Two statics in (**A**) eyes open and (**B**) eyes closed; two dynamics in (**C**) ML weight-shifting exergame, and (**D**) AP weight-shifting exergame. Grey and black arrows show the mediolateral and anteroposterior displacement, respectively. Image credit: Luis Leiva-Cortez, designer. Unpublished figure, created in its original version for this publication.

An AMTI OR6-7 force plate (Watertown, MA, USA), sampling at 100 Hz, was used to obtain the centre-of-pressure (CoP) displacement (measured in cm). This was recorded during (i) eyes open (EO) and (ii) eyes closed (EC) conditions for 60 s each. During these trials, participants were instructed to stand on the force plate with their arms at their sides and their feet shoulder-width apart. CoP displacement was also recorded during (iii) dynamic medial-lateral (ML) weight shifts while playing Penguin (60 s) and during (iv) anterior–posterior (AP) weight shifts while playing Snowboard (30 s). These dynamic assessments were performed with the Nintendo Balance Board placed on top of the force plate. The first two tasks were standard static postural assessments, while the latter two were dynamic postural tasks specifically designed to measure postural control strategies in the mediolateral and antero-posterior planes of motion. All assessments were repeated three times. Figure 2 presents the setup for posturography during all EO, EC, and dynamic trials. For all trials, the CoP data were low-pass filtered at 40 Hz using a second-order Butterworth filter, after which the CoP variables were computed using Python 3.11.13 (Licence released).

#### 2.4.2. Clinical Measures

The 10-Metre Gait Speed Test (GST) was used to assess whether changes in balance control translated to broader motor function, particularly walking speed (m/s). It was conducted at the training site in an unobstructed area that allowed 10 m of steady walking. This test is considered valid and reliable for detecting changes in walking speed over time in older adults [22]. Balance was further assessed using the One-Leg Standing Test (OLS; measured in seconds), a more challenging task. OLS times of less than 10 s have been associated with increased fear of falling and higher fall risk in older adults [23].

### 2.5. Intervention Modalities

Participants in both groups completed the same exergame therapy programme using a Nintendo Wii and Wii Balance Board. The intervention consisted of 18 sessions, each lasting 25 min, delivered over six weeks at a frequency of three times per week.

The programme included three sets of exercises targeting balance control across all planes of motion. The first and second sets used Snowboard, Penguin Slide, and Super Hula Hoop, while the third set featured a Yoga game. A ~2 min seated rest break was provided between sets. In the first set, participants stood in a relaxed posture with arms at their sides; in the second set, the same games were repeated with hands on the waist. The third set involved maintaining a relaxed standing posture during the Yoga game, first with eyes open, then repeated with eyes closed (EC). Figure 2C,D presents examples of the exergames played.

To support adherence, participants in both the TR and FF groups received telephone reminders about upcoming sessions. No modifications were made to the intervention protocols throughout the study.

#### 2.5.1. Telerehabilitation (TR)

**Figure 3:**
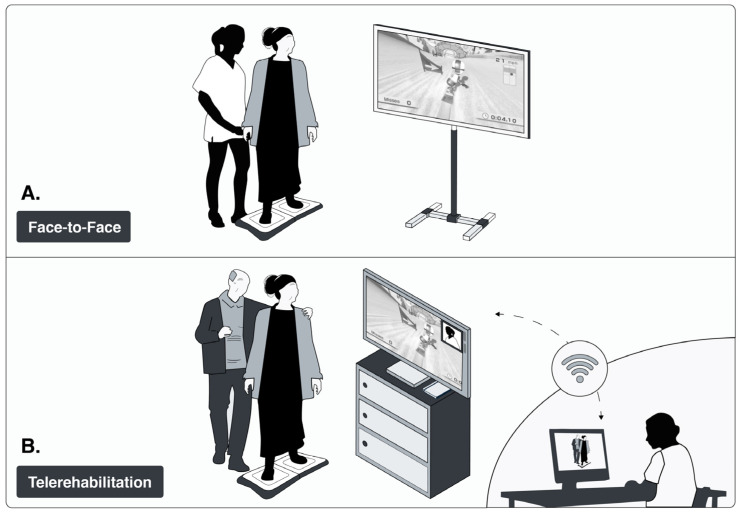
Two Intervention Modalities: (**A**) Face to face and (**B**) Telerehabilitation. Image credit: Luis Leiva-Cortez, designer. Unpublished figure, created in its original version for this publication.

The TR intervention was delivered remotely at a senior citizens’ centre. To facilitate correct session execution and assist with technology use, one participant served as a peer-monitor providing tactile and verbal guidance during the first three weeks to ensure proper exergame performance. During the last 3 weeks, guidance was only verbal. The peer monitor had no clinical or decision-making responsibilities and only provided assistance as instructed and when required. To note, the peer-monitor was previously trained by a physiotherapist about how to provide guidance to participants and operate the exergame system. When assistance was needed, the peer-monitor followed real-time instructions from the physiotherapist, who remained fully responsible for clinical decision-making, feedback, and session progression via remote verbal and visual supervision (Figure 3). Connectivity and technical support were provided by the project team.

#### 2.5.2. Face-to-Face (FF)

The FF exergame sessions were conducted at the Human Motor Control Laboratory of the Universidad de Talca, Talca, Chile. During each session, a physiotherapist provided direct verbal and manual guidance to ensure correct execution of the exergames (Figure 3).

### 2.6. Outcome Measurement

The primary outcome was CoP sway area (CoP*_Sway_*, measured in cm^2^), which has been widely used as a reliable indicator of balance impairment during postural tasks [24,25]. It is defined as the total trajectory of the CoP in the *ML* and *AP* directions. This metric provides a comprehensive assessment of the postural control system’s ability to maintain upright stability, with higher values indicating poorer balance control.

Secondary outcomes included the standard deviation of the CoP (SD_ML_, SD_AP_), which reflects the variability of CoP displacement, and CoP velocity (V_ML_ and V_AP_; measured in cm/s), which reflects the responsiveness of the postural control system to changes in balance. Higher values in both measures are associated with poorer balance control. Specifically, delays in initiating appropriate postural adjustments can lead to greater CoP displacement, requiring rapid compensatory movements to prevent instability [24].

Additionally, the GST and the time in OLS Test were assessed as clinical secondary outcomes.

### 2.7. Statistical Analysis

Descriptive statistics were calculated for all demographic and clinical variables. Between-group comparisons were performed using unpaired t-tests for continuous variables and chi-squared (χ^2^) tests for categorical variables. Assumptions of normality and homogeneity were evaluated using the Shapiro–Wilk and Levene tests, respectively. Our initial plan was to use a two-way repeated-measures ANOVA to analyse the data; however, the assumptions of normality were not met for several outcome variables, as assessed by the Shapiro–Wilk test. Consequently, changes over time within each group were assessed using Friedman’s one-way ANOVA with post hoc pairwise comparisons (Wilcoxon signed-rank test). Between-group comparisons at specific time points (for example, to compare therapeutic effects at week six) were conducted using the Mann–Whitney U test.

Missing data were handled using two complementary methods: (1) replacement with the non-missing weekly mean for each variable, and (2) multiple imputation for missing outcomes and covariates. Both approaches were applied to verify result consistency. A *p*-value ≤ 0.05 was considered statistically significant. All analyses were conducted using a custom Python script (Python version 3.11.13), developed to account for within- and between-group interactions, and publicly available on GitHub (https://github.com/JS-75/AlgosAM; accessed on 13 July 2025).

## 3. Results

### 3.1. Participants

Fifty-five older adults were initially eligible to participate in the study. Of these, 16 were recruited and randomly assigned to either the TR or FF group. The randomly allocated groups were well matched on all demographic characteristics measured in the study (Table 1). The groups also demonstrated homogeneity in their baseline scores for all outcome measure.

Overall, compliance was high at 94%. Only one participant from the TR group had three absences due to personal reasons: a single exergaming session (session 12 in week 4) and two follow-up assessments (in weeks 8 and 10). The missed exergame session coincided with a scheduled assessment for that day. Missing data were addressed using an intention-to-treat analysis (Figure 1).

### 3.2. Primary Outcome

#### 3.2.1. TR Versus FF: Effects at Six Weeks

At the end of the 6-week intervention, CoP*_Sway_*, measured across four postural tasks (two static and two dynamic), showed no significant differences between the study groups. This indicated that the two exergame programmes had similar effects. During the follow-up periods (weeks 8 and 10), the TR group showed a significantly lower CoP*_Sway_* during both, EO (*p* = 0.007) and EC (*p* = 0.037), compared to FF.

#### 3.2.2. Effects of TR and FF over Time

Regarding the effects over time for each study group, the TR group showed a significant decrease in CoP*_sway_* at week 6 compared to baseline, only under the EC condition (*F* = 17.62*, p* = 0.008). In the dynamic postural tasks, a significant reduction in CoP*_sway_* was also observed only under the AP weight shifts trial (Snowboard) at week 6 compared to baseline (*F* = 16.82, *p* = 0.008). However, these significant changes did not persist over time in either trial. For the FF group, significant reductions in CoP*_sway_* were found at week 6 compared to baseline during both EO (*F* = 15.89, *p* = 0.008) and EC conditions (*F* = 14.15*, p* = 0.016). However, these effects did not persist over time. Furthermore, no significant effects over time were found in the primary outcome of the dynamic postural tasks (Table 2).

### 3.3. Secondary Outcome

#### 3.3.1. TR Versus FF: Effects at Six Weeks

The secondary outcomes mirrored the primary outcome’s pattern, with no significant inter-group differences observed in any of the four postural tasks at the six-week mark. Similarly to the primary outcome, during the follow-up period, the TR group demonstrated a significant decrease in secondary outcome variables compared to the FF group; these were observed in the following: At the 8-week, TR demonstrated a significantly lower SD*_ML_* (*p* = 0.04) during EO and lower V*_AP_* (*p* = 0.037) during AP weight shifts. These improvements continued at the 10-week follow-up, with TR showing a significantly lower SD*_ML_* (*p* = 0.01) during EO and lower SD*_ML_* (*p* = 0.04) and V*_ML_* (*p* = 0.001) during AP weight shifts.

At the end of the 6-week intervention period, the TR group demonstrated a significantly greater increase in the GPT compared to the FF group (*p* = 0.01). This effect was consistently maintained until week 10 of follow-up (*p* = 0.002). The OLS analysis revealed no significant differences between groups at the conclusion of the six-week intervention, or during the follow-up period.

#### 3.3.2. Effects of TR and FF over Time

At the conclusion of the six-week intervention, the TR group showed significant reductions in several secondary outcome variables compared to week 0. For the EO postural task, a significant reduction was observed in the SD*_ML_* (*F* = 13.98; *p* = 0.039), with these effects maintained until week 8 (*p* = 0.016). In the EC trial, post hoc analysis showed that significant reductions were also found in SD*_ML_* (*F* = 15.76; *p* = 0.008), SD*_AP_* (*F* = 16.13; *p* = 0.008), and V*_ML_* (*F* = 14.42; *p* = 0.016). The evolution of effects over time on these variables were mixed: (1) SD*_ML_* were maintained decrease at both week 8 (*p* = 0.008) and week 10 (*p* = 0.023), (2) SD*_AP_* showed a notable decrease from week 4 (*p* = 0.008) to week 6 (*p* = 0.008); however, these effects were not maintained during the follow-up period and (3) V*_ML_* exhibited a significant decrease from week 4 (*p* = 0.023) to week 6, with its effects maintained until week 8 (*p* = 0.039) and week 10 (*p* = 0.04). Additionally, at the conclusion of the six-week intervention, the TR group showed significant reductions in both SD*_ML_* and V*_ML_* compared to baseline during the dynamic AP weight shifts postural task (*p* = 0.008 for both variables). Post hoc analysis revealed that V*_ML_* had a significant decrease as early as week 4 (*p* = 0.02), with this effect maintained at the follow-up assessments at week 8 (*p* = 0.04) and week 10 (*p* = 0.04).

At week 6, compared to baseline, FF showed significant reductions in SD*_ML_* (*F* = 16.38; *p* = 0.008), SD*_AP_* (*F* = 16.67; *p* = 0.008) and V*_AP_* (*F* = 15.98; *p* = 0.008) during EO, and significant reductions in SD*_ML_* (*F* = 12.34; *p* = 0.023), SD*_AP_* (*F* = 14.17; *p* = 0.016), V*_ML_* (*F* = 14.68; *p* = 0.016), and V*_AP_* (*F* = 16.48; *p* = 0.008) during EC. Regarding the long-term effects, the FF group also showed a varied response in the EO trial. Post hoc analysis showed that SD*_ML_* significantly decreased from week 4 to week 6 (*p* = 0.008); however, values significantly increased during the follow-up period, returning to baseline levels at week 8 (*p* = 0.039, compared to week 6) and week 10 (*p* = 0.016, compared to week 6). In contrast, SD*_AP_* and V*_AP_* exhibited similar and sustained improvements. Both variables showed significant decreases from week 4 to week 6 (*p* = 0.002 and *p* = 0.01, respectively) and these reductions were maintained throughout the follow-up, with significant decreases observed at week 8 (*p* = 0.01 and *p* = 0.02, respectively) and week 10 (*p* = 0.02 and *p* = 0.04, respectively). The FF group’s EC postural task revealed distinct patterns regarding long-term effects on secondary outcomes. While both SD*_ML_* and SD*_AP_* showed a significant decrease from week 4 to week 6 (both *p* = 0.008), these variables significantly increased during the follow-up period, returning to near baseline levels. Specifically, SD*_ML_* increased by week 10 (*p* = 0.04) and SD*_AP_* by week 8 (*p* = 0.04). In contrast, the CoP velocity variables, V*_ML_* and V*_AP_*, exhibited more sustained improvements. Both showed a significant decrease at week 4 (both *p* = 0.02) that was maintained through week 6 and continued into the follow-up period, with effects lasting until week 10 (*p* = 0.023) and week 8 (*p* = 0.008), respectively. Finaly, SD*_ML_* and V*_ML_* both showed a significant decrease in the dynamic ML weight shifts postural task from week 4 (both *p* = 0.02) until week 6 of the intervention. However, only V*_ML_* maintained a significant decrease over time until week 8 (*p* = 0.04) (Table 2).

From the evolution of effects over time in the GST, TR showed significantly faster speed from week 2 (*p* = 0.03); however, the greatest increase occurred at week 6, which was then maintained until week 10. For the FF, compared to baseline, a significant speed increase was found by the end of the intervention at week 6 (*F* = 13.89; *p* = 0.016) in the GST. This improvement, however, effaced during the follow-up period, at weeks 8 (*p* = 0.016) and week 10 (*p* = 0.039) when compared to baseline (Table 3).

When analysing the changes over time in OLS for each group, only the TR demonstrated significant improvements by the intervention’s conclusion. TR showed a significant improvement by week 6, where times were increased for both sides, left (*p* = 0.04) and right standing (*p* = 0.04), when compared to baseline. This was maintained until week 8 of follow-up, compared to baseline, for the left side (*p* = 0.008) (Table 3).

Adverse events: No adverse effects were reported in any group at any stage.

## 4. Discussion

This randomised controlled trial is the first, to our knowledge, to compare the effectiveness of a low-cost exergame programme using the Nintendo Wii Balance Board delivered via TR versus FF physiotherapy in older adults. Both modalities demonstrated comparable effectiveness in improving postural control, as measured using posturography and validated clinical tests. Importantly, this study also examined the persistence of these effects over time, offering insights into the sustainability of each approach.

Our findings confirmed that a six-week exergame intervention delivered through either TR or FF was effective in enhancing postural control. Both groups showed improvements in primary and secondary outcomes, including CoP*_Sway_* measures and the OLS. These results support the use of Wii-based rehabilitation to target balance deficits in older adults and demonstrate that TR is a viable and scalable alternative to traditional FF delivery.

A notable observation was the TR group’s superior performance in gait speed from the second week of the intervention through to follow-up. Although there was a non-significant decrease in speed at week 10, values remained above baseline. We hypothesise that this improvement may be partly due to the familiar environment in which the TR group received the intervention and underwent testing. This comfort may have reduced anxiety and enhanced performance, a phenomenon supported by prior studies showing that therapeutic engagement in familiar settings, such as the home [26] or community centres, can positively affect balance and mobility outcomes in older adults [6,27].

The TR group also demonstrated a greater ability to maintain improvements in postural control post-intervention, particularly during the most challenging dynamic postural tasks. Significant reductions in CoP*_Sway_* and variability under EC and dynamic AP weight-shift conditions were observed, suggesting more robust neuromotor adaptation. These findings may be partially attributed to the consistent support provided by a peer monitor, an older adult participant trained to guide the sessions under remote physiotherapist supervision. This peer-led model likely enhanced confidence and compliance [6], creating a positive therapeutic dynamic within the TR group. Importantly, the peer monitor had no decision-making or therapeutic responsibilities and provided assistance only when needed and under real-time physiotherapist instruction, ensuring that the intervention remained physiotherapist-led throughout.

In contrast, the FF group showed improvements during the intervention but tended to return to baseline or early-intervention levels by the follow-up period. This decline was evident in variables such as SD*_ML_* under both EO and EC conditions. These differences suggest that TR may be better suited for sustaining gains in certain aspects of postural control over time.

Importantly, both modalities demonstrated task-specific benefits. The FF group showed greater improvements in ML weight-shift tasks, modifying two secondary outcomes, while the TR group showed superior performance in AP weight-shift tasks, modifying both primary and secondary outcomes. Notably, only the TR group reduced CoP sway in dynamic conditions, indicating a potential for greater core or trunk muscle engagement, an effect associated with improved gait speed and functional stability in older adults [28].

The feedback-rich nature of exergames, which combines real-time visual and auditory cues, may have contributed to these improvements by enhancing motor learning and engagement. Weight-shift games, such as those used in this study, promote active postural strategies, requiring participants to make frequent and adaptive adjustments. These interactive elements may help explain why both TR and FF modalities effectively targeted key aspects of postural control, albeit through slightly different mechanisms.

Our results are consistent with previous research demonstrating the effectiveness of TR for improving balance and functional outcomes in older adults and individuals with neurological conditions. Truijen et al. (2022) [18], Jirasakulsuk et al. (2022) [29], and Deshmukh (2023) [30] all reported similar findings regarding the similar effects of TR compared to conventional physiotherapy. Other studies, such as those by Su (2023) [31] and Lee (2024) [32], highlight the need to tailor telerehabilitation tools to the type of tasks being trained, i.e., static or reactive, a consideration our task-specific results support.

The complementary nature of TR and FF observed in our study suggests that these modalities need not be seen as mutually exclusive. Rather, they can be delivered sequentially or in combination to maximise clinical outcomes. For example, TR may serve as a first-line approach to establish and maintain postural improvements, particularly in community settings with limited access to in-person care. This is due to its demonstrable short- and medium-term effects. Subsequently, and within a clinical rehabilitation environment, FF interventions could then be introduced to further target specific balance domains, such as those involving more reactive tasks.

### Strengths and Limitations of the Study

This study has several strengths, including the use of validated objective and clinical outcome measures, a blinded and controlled design, and a novel comparison of two scalable intervention modalities. Nevertheless, a major strength was its comprehensive approach to assessing postural control. While a reduction in CoP*_sway_* following an intervention is known to indicate improved postural control, a comprehensive interpretation required considering both gold-standard laboratory measurements—such as posturography—and clinical assessments. Our analysis integrated both primary and secondary CoP outcomes alongside clinical test results, which collectively provided a complete assessment of postural control. This multifaceted approach was a key strength of the study, enabling a more robust interpretation of the intervention’s effects. However, our study has some limitations that need to be acknowledged. These include the small sample size and setting restricted to a single region in Chile. Additionally, the use of the Nintendo Wii, while cost-effective, may not fully replicate the capabilities of more advanced and newer exergame systems. This presents a challenge in the technological development of low-cost and effective devices for this population, which is currently being addressed by this team of researchers [26]. Our sample consisted of relatively high-functioning older adults, which improved adherence and reduced health- and function-related confounders but may limit generalizability to less mobile populations. Nevertheless, older adults with subtle postural control decline may still benefit from interventions that prevent further deterioration and reduce fall risk.

## 5. Conclusions

This trial demonstrates that low-cost exergame therapy delivered via TR with a peer-monitor is as effective as FF delivery in improving postural control in older adults. The findings highlight the potential for TR to sustain balance control gains over time and support its integration into community-based physiotherapy services. Rather than competing, TR and FF may serve as complementary tools to meet the growing demand for accessible, effective rehabilitation for ageing populations.

## Figures and Tables

**Table 1:** Baseline Characteristic of the Participants.

Characteristics	TR (*n* = 8)	FF (*n* = 8)	*p*-Value
Age (y), mean (SD)	69 (2)	69.3 (3.1)	0.529
Sex, *n* (%)			
Female	6 (75)	7 (87.5)	0.456
Male	2 (25)	1 (12.5)	0.679
Weight (kg), mean (SD)	70 (7.9)	67.8 (7.8)	0.498
Height (m), mean (SD)	1.6 (0.8)	1.6 (0.1)	0.643
BMI (kg/m^2^), mean (SD)	26.8 (2.8)	27.5 (3.4)	0.751
Cognitive function (MMSE, 0 to 30), mean (SD) ^a^	26.2 (2.3)	26.5 (2.2)	0.435
Functional capacity (SPPB, 0 to 12), mean (SD) ^a^	9.7 (1.5)	9.6 (1.6)	0.869

Data shown as *n* (%) or mean ± SD, TR = telerehabilitation group, FF = face to face group, MMSE = Mini Mental State Examination, SPPB = Short Physical Performance Battery, kg = kilogram, m = metre, m^2^ = square metre. ^a^ Higher scores indicate better function.

**Table 2:** Outcome Measures During Four Postural Tasks for Both Groups Over Time.

				Baseline	Mid-Intervention		End of Intervention	Follow-Ups		
				Week 0	Week 2	Week 4	Week 6	Week 8	Week 10	
	Measure	Task	Group	Median [IQR]	Median [IQR]	Median [IQR] ^a^	Median [IQR]	Median [IQR] ^a^	Median [IQR] ^a^	*p*-Value ^b^
**Primary Outcome**	**CoP***sway* **(cm^2^)**	EO	FF	9.81 [6.84−11.41]	8.24 [5.88−10.74]	8.12 [6.95−15.50]	4.90 [4.21−6.52]	6.33 [5.64−8.48]	6.76 [6.36−9.81]	0.021
			TR	7.72 [6.89−10.44]	6.11 [5.12−8.64]	7.36 [6.20−16.76]	5.68 [3.59−8.19]	4.67 [3.31−5.98]	4.47 [3.35−5.60]	0.005
		EC	FF	13.22 [9.93−15.32]	10.53 [6.03−15.26]	11.50 [10.30−12.99]	5.79 [4.55−7.41]	8.24 [5.93−10.92]	8.45 [6.97−11.22]	0.002
			TR	13.80 [8.72−24.03]	11.16 [7.89−17.74]	10.92 [6.47−12.50]	6.20 [4.46−8.90]	5.64 [3.40−6.07]	7.55 [4.15−8.25]	0.000
		ML	FF	1402.27 [1180.19−1520.95]	1155.52 [885.47−1588.96]	1210.73 [862.24−1716.70]	813.08 [748.56−1098.04]	955.83 [756.81−1196.81]	932.38 [802.84−1145.87]	0.028
			TR	845.71 [538.81−1153.84]	918.25 [859.19−1138.38]	961.01 [890.74−1154.89]	916.79 [794.21−1038.10]	801.37 [778.72−998.42]	959.37 [831.64−993.80]	0.279
		AP	FF	603.24 [496.70−965.90]	846.91 [522.10−1289.32]	850.46 [732.39−1161.67]	661.63 [538.96−988.81]	564.93 [437.83−786.33]	614.27 [525.42−933.93]	0.013
			TR	704.70 [552.93−768.26]	716.85 [631.96−1071.65]	677.26 [504.94−802.74]	427.75 [385.20−591.44]	435.00 [340.65−656.97]	448.04 [377.14−611.11]	0.002
**Secondary Outcome**	**SD***ML* **(cm)**	EO	FF	0.17 [0.15−0.20]	0.19 [0.17−0.21]	0.17 [0.15−0.30]	0.13 [0.10−0.14]	0.15 [0.13−0.21]	0.18 [0.15−0.22]	0.010
			TR	0.22 [0.18−0.24]	0.19 [0.17−0.23]	0.15 [0.13−0.25]	0.15 [0.12−0.17]	0.12 [0.10−0.12]	0.11 [0.10−0.13]	0.002
		EC	FF	0.23 [0.18−0.26]	0.19 [0.16−0.24]	0.19 [0.18−0.22]	0.12 [0.10−0.14]	0.15 [0.11−0.17]	0.17 [0.14−0.20]	0.001
			TR	0.25 [0.20−0.29]	0.19 [0.17−0.23]	0.20 [0.13−0.23]	0.14 [0.10−0.15]	0.11 [0.10−0.13]	0.14 [0.12−0.17]	0.000
		ML	FF	7.89 [7.53−8.95]	7.22 [6.73−10.06]	7.91 [6.55−10.01]	6.03 [5.80−6.70]	6.91 [5.42−7.83]	6.87 [6.16−7.79]	0.024
			TR	5.38 [4.90−7.10]	6.82 [6.55−7.87]	6.86 [6.75−7.32]	6.81 [6.53−6.95]	6.77 [6.21−7.13]	6.88 [6.32−7.32]	0.261
		AP	FF	3.41 [1.92−4.21]	3.11 [2.85−3.88]	3.52 [2.96−4.47]	2.68 [2.00−3.78]	2.40 [1.73−3.08]	3.47 [2.29−4.00]	0.335
			TR	2.66 [2.40−3.32]	3.04 [2.80−3.59]	2.58 [2.39−2.90]	1.95 [1.78−2.35]	1.96 [1.47−2.43]	2.05 [1.66−2.23]	0.000
	**SD***AP* **(cm)**	EO	FF	0.48 [0.44−0.56]	0.41 [0.39−0.43]	0.45 [0.38−0.48]	0.31 [0.28−0.36]	0.35 [0.32−0.37]	0.34 [0.32−0.36]	0.000
			TR	0.37 [0.34−0.48]	0.39 [0.34−0.41]	0.44 [0.38−0.61]	0.37 [0.33−0.39]	0.32 [0.29−0.34]	0.33 [0.29−0.35]	0.005
		EC	FF	0.57 [0.47−0.63]	0.51 [0.43−0.54]	0.52 [0.47−0.59]	0.39 [0.34−0.43]	0.42 [0.40−0.46]	0.40 [0.37−0.42]	0.023
			TR	0.57 [0.46−0.71]	0.58 [0.45−0.77]	0.55 [0.41−0.62]	0.42 [0.38−0.47]	0.38 [0.31−0.43]	0.41 [0.35−0.45]	0.000
		ML	FF	1.59 [1.24−1.76]	1.46 [1.33−1.67]	1.48 [1.23−1.61]	1.13 [1.07−1.29]	1.19 [1.05−1.32]	1.21 [1.02−1.30]	0.049
			TR	1.25 [1.04−1.50]	1.25 [1.19−1.45]	1.40 [1.31−1.63]	1.17 [1.01−1.34]	1.12 [1.02−1.18]	1.15 [1.03−1.30]	0.003
		AP	FF	2.32 [1.98−4.05]	3.02 [2.17−3.81]	3.60 [2.95−3.97]	3.18 [2.76−3.75]	3.01 [2.42−3.37]	2.91 [2.23−3.41]	0.689
			TR	2.69 [2.26−3.34]	3.05 [2.63−3.54]	2.93 [2.56−3.24]	2.60 [2.53−2.77]	2.64 [2.34−2.93]	2.68 [2.60−2.87]	0.299
	**V***ML* **(cm/s)**	EO	FF	0.41 [0.35−0.46]	0.37 [0.35−0.42]	0.42 [0.33−0.49]	0.31 [0.24−0.37]	0.32 [0.23−0.34]	0.33 [0.28−0.37]	0.000
			TR	0.40 [0.31−0.66]	0.38 [0.26−0.46]	0.47 [0.32−0.56]	0.38 [0.25−0.45]	0.30 [0.24−0.42]	0.26 [0.25−0.41]	0.001
		EC	FF	0.51 [0.46−0.59]	0.42 [0.36−0.52]	0.46 [0.38−0.52]	0.36 [0.29−0.37]	0.37 [0.27−0.40]	0.37 [0.31−0.37]	0.000
			TR	0.55 [0.37−0.64]	0.59 [0.33−0.66]	0.45 [0.35−0.63]	0.43 [0.29−0.53]	0.36 [0.29−0.51]	0.33 [0.26−0.55]	0.000
		ML	FF	18.82 [15.02−21.01]	17.91 [11.98−23.65]	15.23 [10.91−21.60]	12.29 [8.91−16.07]	13.58 [10.74−17.26]	13.32 [10.10−16.64]	0.000
			TR	13.67 [12.01−16.14]	14.23 [13.64−14.77]	14.57 [13.73−15.15]	12.81 [12.25−13.62]	11.79 [10.91−12.95]	12.23 [11.04−12.89]	0.027
		AP	FF	7.99 [5.36−8.98]	7.40 [6.70−9.88]	8.25 [7.01−11.93]	6.41 [5.89−7.78]	6.00 [5.54−6.21]	7.72 [6.72−8.24]	0.016
			TR	7.04 [5.60−8.49]	7.15 [6.20−7.76]	6.84 [5.52−7.80]	4.65 [3.80−6.06]	4.50 [3.63−6.20]	4.97 [4.06−5.61]	0.001
	**V***AP* **(cm/s)**	EO	FF	0.82 [0.74−0.90]	0.81 [0.69−0.86]	0.78 [0.77−0.96]	0.66 [0.63−0.68]	0.61 [0.59−0.64]	0.67 [0.61−0.78]	0.001
			TR	0.80 [0.62−0.91]	0.81 [0.74−0.88]	0.93 [0.86−1.21]	0.78 [0.67−0.82]	0.75 [0.67−0.78]	0.65 [0.53−0.76]	0.001
		EC	FF	1.05 [0.92−1.26]	1.05 [0.78−1.24]	1.06 [0.96−1.22]	0.86 [0.74−1.03]	0.90 [0.85−1.01]	0.92 [0.81−1.04]	0.000
			TR	1.08 [0.83−1.51]	1.31 [1.02−1.51]	1.13 [0.98−1.42]	1.03 [0.81−1.30]	0.97 [0.85−1.20]	0.93 [0.70−1.12]	0.000
		ML	FF	4.48 [3.96−4.88]	4.60 [3.42−6.57]	4.64 [3.14−6.62]	3.96 [2.72−5.16]	4.12 [2.85−5.14]	3.69 [3.18−4.89]	0.093
			TR	3.87 [3.28−4.65]	4.01 [3.89−4.38]	4.98 [4.40−5.50]	4.53 [4.10−4.65]	3.84 [3.59−4.55]	3.96 [3.49−4.90]	0.181
		AP	FF	5.65 [5.23−10.37]	6.84 [5.52−11.33]	8.83 [6.81−10.62]	8.33 [5.96−10.29]	7.97 [6.56−9.28]	6.38 [5.54−10.37]	0.112
			TR	6.21 [4.73−7.57]	6.64 [5.49−9.22]	6.91 [6.31−7.22]	5.76 [5.29−6.85]	5.63 [5.40−5.80]	5.38 [5.28−6.01]	0.061

^a^ Missing values from only an older adult on three occasions: fourth week and at follow-up during the eighth and tenth weeks, respectively (TR group, *n* = 1 on three times); ^b^ Friedman’s one-way ANOVA. IQR: interquartile range; CoP: centre-of-pressure; *ML*: medial-lateral; *AP*: anterior–posterior; CoP*_Sway_*: area of CoP sway; SD*_ML_* and SD*_AP_*: standard deviation of CoP in the directions medial-lateral and anterior–posterior; V*_ML_* and V*_AP_*: CoP velocity in the directions medial-lateral and anterior–posterior. cm: centimetres; cm/s: centimetres/seconds and cm^2^: square centimetres. Task, EO: eyes-open; EC: eyes-closed; ML: ML weight-shifting exergame, and AP: AP weight-shifting exergame. Group, FF: face to face and TR: telerehabilitation.

**Table 3:** Outcome Measures During Two Clinical Tests for Both Groups Over Time.

		Baseline	Mid-Intervention		End of Intervention	Follow-Ups		
		Week 0	Week 2	Week 4	Week 6	Week 8	Week 10	
Clinical Test	Group	Median [IQR]	Median [IQR]	Median [IQR] ^a^	Median [IQR]	Median [IQR] ^a^	Median [IQR] ^a^	*p*-Value ^b^
**Gait Speed (m/s)**	FF	0.97 [0.96−1.01]	1.04 [0.99−1.05]	1.04 [1.02−1.27]	1.04 [1.00−1.09]	1.03 [0.99−1.06]	0.98 [0.97−1.01]	0.075
	TR	1.23 [1.06−1.37]	1.25 [1.17−1.42]	1.16 [1.10−1.24]	1.20 [1.09−1.38]	1.29 [1.11−1.39]	1.21 [1.11−1.33]	0.099
**OLS right (s)**	FF	8.17 [6.42−16.00]	9.17 [3.67−21.17]	12.67 [6.42−18.08]	14.00 [7.50−22.33]	14.67 [9.67−17.83]	10.17 [8.00−14.33]	0.535
	TR	10.33 [5.17−19.25]	11.50 [6.33−17.00]	15.60 [11.42−21.00]	14.67 [9.00−24.00]	14.20 [7.00−19.50]	15.10 [14.20−17.00]	0.234
**OLS Left (s)**	FF	14.67 [3.83−19.50]	8.83 [4.08−16.42]	9.50 [5.75−16.92]	13.50 [8.25−17.75]	12.50 [10.08−16.25]	12.00 [7.92−16.00]	0.212
	TR	10.00 [4.25−17.67]	8.50 [6.58−16.08]	12.16 [9.08−16.00]	14.67 [12.25−24.75]	12.65 [8.98−15.50]	12.65 [11.91−16.75]	0.005

^a^ Missing values from only an older adult on three occasions: fourth week and at follow-up during the eighth and tenth weeks, respectively (TR group, *n* = 1 on three times); ^b^ Friedman’s one-way ANOVA. IQR: interquartile range. m: metres; m/sec: metres/seconds; and s: seconds. OLS: One-Leg Standing Test. Group, FF: face to face and TR: telerehabilitation.

## Data Availability

The data presented in this study are available on request from the corresponding author due to privacy or ethical restrictions.

## Abbreviations

The following abbreviations are used in this manuscript:

TR	Telerehabilitation
FF	Face-to-face
CoP	Centre-of-pressure
EO	Eyes Open
EC	Eyes Closed
ML	Medial-lateral
AP	Anterior–posterior
GST	Gait Speed Test
OLS	One-Leg Standing Test
CoP_Sway_	CoP sway area
SD_ML_	Standard deviation of the CoP medial-lateral
SD_AP_	Standard deviation of the CoP anterior–posterior
V_ML_	Velocity of the CoP medial-lateral
V_AP_	Velocity of the CoP anterior–posterior
MMSE	Mini Mental State Examination
SPPB	Short Physical Performance Battery
